# Homology of the cranial vault in birds: new insights based on embryonic fate-mapping and character analysis

**DOI:** 10.1098/rsos.160356

**Published:** 2016-08-10

**Authors:** Hillary C. Maddin, Nadine Piekarski, Elizabeth M. Sefton, James Hanken

**Affiliations:** Museum of Comparative Zoology, Harvard University, 26 Oxford Street, Cambridge, MA 02138, USA

**Keywords:** cranial neural crest, mesoderm, skull, evolution, axolotl, transgenic

## Abstract

Bones of the cranial vault appear to be highly conserved among tetrapod vertebrates. Moreover, bones identified with the same name are assumed to be evolutionarily homologous. However, recent developmental studies reveal a key difference in the embryonic origin of cranial vault bones between representatives of two amniote lineages, mammals and birds, thereby challenging this view. In the mouse, the frontal is derived from cranial neural crest (CNC) but the parietal is derived from mesoderm, placing the CNC–mesoderm boundary at the suture between these bones. In the chicken, this boundary is located within the frontal. This difference and related data have led several recent authors to suggest that bones of the avian cranial vault are misidentified and should be renamed. To elucidate this apparent conflict, we fate-mapped CNC and mesoderm in axolotl to reveal the contributions of these two embryonic cell populations to the cranial vault in a urodele amphibian. The CNC–mesoderm boundary in axolotl is located between the frontal and parietal bones, as in the mouse but unlike the chicken. If, however, the avian frontal is regarded instead as a fused frontal and parietal (i.e. frontoparietal) and the parietal as a postparietal, then the cranial vault of birds becomes developmentally and topologically congruent with those of urodeles and mammals. This alternative hypothesis of cranial vault homology is also phylogenetically consistent with data from the tetrapod fossil record, where frontal, parietal and postparietal bones are present in stem lineages of all extant taxa, including birds. It further implies that a postparietal may be present in most non-avian archosaurs, but fused to the parietal or supraoccipital as in many extant mammals.

## 1. Introduction

The cranial vault forms the roof of the vertebrate skull. In bony fishes, the cranial vault comprises numerous irregularly arranged bony plates, but during the evolution of tetrapods the number of separate bones is reduced and those that remain become organized into a regular and consistent pattern [1,2]. In primitive tetrapods, such as *Acanthostega*, the cranial vault consists of a longitudinal series of paired bones. From rostral to caudal, these are the frontals, the parietals and the postparietals [1].

The homology of these bones, as implied by their names, is well accepted for most tetrapods, even when the bones fuse in various combinations. Among amphibians, the cranial vault of salamanders and caecilians includes separate frontals and parietals, whereas in frogs these bones are considered by most to be fused into a single or paired frontoparietal [3,4] (but see [5–9]). Discrete postparietals are not seen in the adult skull of any living amphibian. They are regarded as lost in all three extant groups, or alternatively that they are fused to the parietals in some or all species [10,11]. In mammals, the vault retains a full complement of bones—frontals, parietals and interparietal(s); the latter is considered a fusion of the primitively discrete postparietals with the laterally adjacent tabulars [12]. The vault of all reptile lineages includes frontals and parietals. Living members of these lineages are widely considered to have lost postparietals, seemingly independently [13], although there are accounts of postparietals in some crocodylian embryos [1,14].

Recent reports, however, challenge the implied homology of bones of the cranial vault in birds relative to those of other tetrapods, and even other amniotes. Traditionally, the avian cranial vault is considered derived from the primitive tetrapod pattern through enlargement of the frontals, retention of the parietals and loss of the postparietals [1,2]. Alternatively, several authors have proposed instead that the avian ‘frontal’ represents a fusion between frontal and parietal bones of other tetrapods and should, therefore, be called a frontoparietal, and that the avian ‘parietal’ is more appropriately regarded as a postparietal^1^ [12,15,16]. Support for this alternative hypothesis comes primarily from fate-mapping and genetic-labelling studies that resolve the embryonic origin of these bones in the chicken and mouse. The embryonic origin of skull bones is generally regarded as highly conserved among vertebrates [17], and developmental data are routinely used for assessing bone homology [12,18]. In the chicken, the anterior portion of the frontal is derived from cranial neural crest (CNC), whereas the posterior portion is derived from mesoderm [16,19]. In the mouse, however, the frontal is derived from CNC [20]. The parietal is derived from mesoderm in both species. There are two possible explanations for this discrepancy: either the developmental origin of homologous cranial bones is variable among amniotes, or currently accepted homologies for bones of the cranial vault in birds or mammals, and possibly other tetrapods, are incorrect [15].

Resolution of this disagreement regarding the homology of the avian cranial vault requires comparative data from additional tetrapod taxa. Here, we evaluate the homology of the bones of the cranial vault based on developmental, topologic and phylogenetic criteria, including a newly derived embryonic fate map for a non-amniote tetrapod, the Mexican axolotl (*Ambystoma mexicanum*). Our data indicate extreme conservation of the embryonic origin of the bony skull: developmental patterns in the axolotl, mouse and chicken are virtually identical. This result provides additional support for previous claims that long-accepted homologies for bones of the avian cranial vault are incorrect and that the two principal bones, currently named frontal and parietal, may be misidentified. It further supports the alternative hypothesis of cranial vault homology outlined above, viz. frontoparietal and postparietal. We discuss the implications of this alternative hypothesis in a broader evolutionary context, including those for the evolution of the cranial vault in archosaurs, which comprise the common ancestor of crocodylians and birds and all its descendants, including dinosaurs.

## 2. Material and methods

### 2.1. Animals and husbandry

We employed a transgenic line of axolotl that ubiquitously expresses green fluorescent protein (GFP) and has been successfully used for long-term fate mapping [21,22]. Embryos were obtained from the Hanken laboratory colony at the Museum of Comparative Zoology and from the *Ambystoma* Genetic Stock Center at the University of Kentucky. Prehatching embryos were staged [23] and maintained in 40% Holtfreter solution (HFT). Posthatching larvae were staged [24] and maintained in 20% HFT.

### 2.2. Cranial neural crest and mesoderm transplantations

In preparation for transplantation, the jelly coat was manually removed from late-gastrula embryos by using forceps. All surgeries were performed on the left side; the right side served as an internal control. In general, segments of CNC or cranial mesoderm were transplanted from GFP-positive donor embryos into stage-matched, wild-type hosts, following the methods of Piekarski *et al*. [25]. Three sets of transplantation experiments were carried out: neural fold transplants, neural crest stream transplants and mesoderm transplants.

### 2.3. Cranial neural crest stream terminology

CNC in all vertebrates, including agnathans, comprises three principal populations of migratory cells that emerge from different rostrocaudal positions along the developing brain [26–29]. Despite this basic similarity among taxa, these populations are frequently assigned different names by different authors in an attempt to convey the particular migratory pathway(s) followed and/or cranial region occupied by each population—features that may vary somewhat among species. We use ‘mandibular stream’ in reference to the rostralmost of the three principal populations in the axolotl, whose cells migrate anterior and posterior to the eye and populate the first (mandibular) oropharyngeal arch [30]. It is equivalent to the ‘trigeminal’ neural crest [26,27], which may comprise distinct preoptic, postoptic and mandibular-arch streams [31,32]. We use ‘hyoid stream’ in reference to the second principal population, which populates the second (hyoid) arch. We use ‘branchial stream’ in reference to the third population (‘circumpharyngeal crest’ [33]), which contributes to more posterior arches (in axolotl, arches 3–6).

### 2.4. Histology and immunohistochemistry

To confirm the timing of ossification and the morphology of individual bones, unoperated axolotl larvae were prepared as cleared whole mounts differentially stained for bone and cartilage with alizarin red S and Alcian blue 8GX, respectively [34]. Chimeras were reared for three to six months, by which time most skull bones have developed, and then immersed in aqueous MS-222 (tricaine methanesulfonate; Sigma-Aldrich, St. Louis, MO, USA), rinsed in phosphate-buffered saline (PBS), and fixed in 4% paraformaldehyde (PFA) at 4°C for 48 h.

Chimeras were analysed histologically and immunohistochemically using methods described in Piekarski *et al*. [25]. See the electronic supplementary materials for details.

### 2.5. Analysis

For fate-mapping individual CNC streams and mesoderm, 57 animals were analysed. Bone labelling was examined in serial transverse sections. In our sections, GFP labelling is restricted to relatively few cells in each bone. Most labelled cells occupy the outer proliferation zones, and especially the periosteum. See the electronic supplementary material, figure S1, for the entire fate map of the axolotl cranium.

## 3. Results

We documented the contributions of both CNC and cranial mesoderm to individual skull bones in the axolotl by transplanting GFP-labelled donor cells into wild-type hosts. With one exception (see below, §3.2), our results are directly comparable with detailed fate maps for the chicken, insofar as they differentiate CNC from mesodermal contributions as well as those from individual CNC streams. Our results are more generally comparable with those for the mouse, which only differentiate CNC from mesodermal contributions.

Different research groups have derived different fate maps for the chicken, despite having used the same quail-chick chimeric system to label both CNC and mesoderm. Their results differ, in particular, with respect to the location of the CNC–mesoderm boundary in the cranial vault [35]. The boundary extends transversely through the middle of the frontal bone according to Noden [36] and Le Lièvre [37], whereas Couly *et al*. [38] locate it more posteriorly, along the suture between the parietal and supraoccipital. We compare our axolotl data to the former results [36,37], which have been validated in subsequent studies that use the same and alternative labelling techniques [19,39] and which constitute a growing consensus [33].

### 3.1. The cranial vault in the axolotl

The cranial vault in the axolotl consists of paired frontal and parietal bones, which begin to ossify at stages 50/51 [20], 10–15 days after hatching [40]. By stage 53, both bones have increased in size but have not yet attained their maximum width. CNC and mesodermal contributions were assessed at stages 53 and higher.

**Figure 1.**
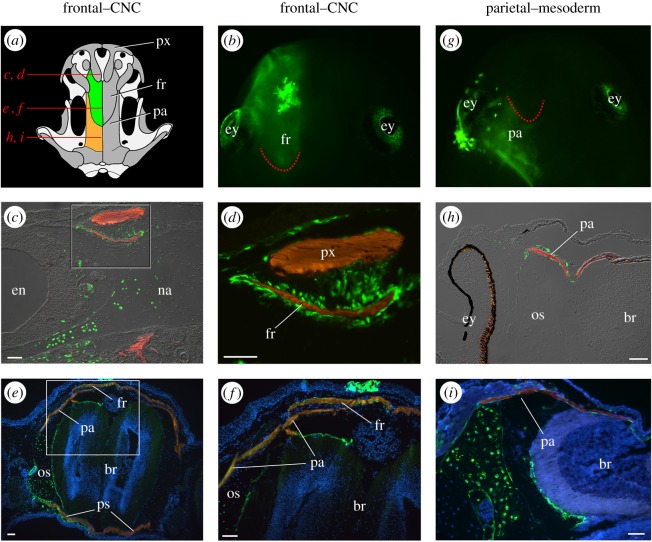
In axolotl, the frontal bone is derived from mandibular-stream cranial neural crest (CNC) and the parietal bone is derived from mesoderm. (*a*) Axolotl skull in dorsal view; left frontal is green, left parietal is orange, other bones are dark grey, and cartilage is light grey. The frontal (fr) is overlapped rostrally by the premaxilla (px) and overlaps the parietal (pa) caudally. Red lines indicate the planes of sections depicted in panels (*c–f*)*,* (*h*) and (*i*). (*b*) Juvenile axolotl that as a neurula received a unilateral (left) graft of GFP-labelled mandibular-stream CNC. Dorsal view, anterior is at the top. The bright area overlying the left frontal is a patch of labelled epidermis; the retina of the eye (ey) is autofluorescent, but not labelled. The dotted red line indicates the caudal margin of the frontal. (*c*–*f*) The frontal bone is labelled in transverse sections of juvenile axolotls. Boxed areas in left panels are shown at higher magnification on the right; GFP-positive cells are green, bone is red, nuclei are blue. (*c*,*d*) Numerous GFP-positive cells surround the frontal in a section just anterior to the eye. (*e,f*) GFP-positive cells invest the frontal in a section at the level of the eye. The bone is largely acellular; labelled cells populate the surrounding periosteum. (*g*) Juvenile axolotl that as a neurula received a unilateral (left) graft of cranial paraxial mesoderm. Dorsal view, anterior is at the top. The dotted red line indicates the caudal margin of the frontal bone. (*h*,*i*) Transverse sections of juvenile axolotls show GFP labelling of the parietal bone. (*h*) GFP-positive cells surround the parietal anteriorly. (*i*) GFP-positive cells are present in the periosteum that surrounds the parietal posteriorly. Scale bars equal 100 µm. br, brain; en, external naris; na, nasal cartilage; os, orbitosphenoid; ps, parasphenoid.

The frontal bone is labelled in 3 of 12 animals that received an embryonic transplant of mandibular-stream CNC (figure 1*a,b*). Labelling is found throughout the bone's entire length but is most intense along its rostral and caudal edges, which presumably are sites of active growth. Labelled cells are located mainly in the periosteum. The premaxilla, which also is derived from the mandibular stream, overlaps the frontal bone rostrally (figure 1*a*,*c*,*d*), whereas the lateral edge of the frontal overlaps the parietal caudally (figure 1*a*). Labelled cells are visible in the caudal region of the frontal but never in the adjacent parietal (figure 1*c–f*). There is no labelling of either the frontal or the parietal following any transplantation of hyoid or branchial stream CNC.

The parietal bone is labelled in 10 of 45 animals that received an embryonic transplant of cranial paraxial mesoderm (figure 1*g*–*i*). Labelled cells are present primarily in the periosteum. Mesoderm from the region dorsal to the mandibular arch contributes to the anterior portion of the parietal, while mesoderm dorsal to the hyoid and anterior branchial arches contributes to its posterior portion.

### 3.2. Axolotl and amniote fate maps

The cranial fate map for the axolotl is extremely similar to those for the chicken and mouse with respect to territories derived from CNC versus mesoderm. Rostral bones, including both upper and lower jaws, the snout and rostral portions of both the palate and the cranial vault, are derived entirely from CNC, as is the jaw suspensorium. Mesodermal contributions are restricted to caudal portions of the cranial vault and palate, as well as the occipital region.

**Figure 2.**
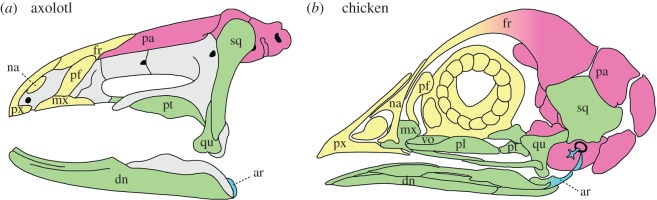
Skulls of (*a*) axolotl and (*b*) chicken depicting contributions from CNC and mesoderm. Mandibular-stream CNC gives rise to most bones of the facial region, including similar respective contributions from its anterior (yellow) and posterior (green) portions, whereas hyoid stream CNC (blue) contributes to the posterior tip of the articular bone in the lower jaw and to the stapes (not visible in axolotl in lateral view). The branchial stream does not contribute to the skull proper in either species. Mesoderm-derived bones are pink. Diagram in (*b*) is based on [41]. ar, articular; dn, dentary; fr, frontal; mx, maxilla; na, nasal; pa, parietal; pf, prefrontal; pl, palatine; pt, pterygoid; px, premaxilla; qu, quadrate; sq, squamosal; vo, vomer.

A more detailed comparison of fate maps between the axolotl and chicken reveals that this extreme similarity extends to the respective contributions of different CNC streams, and even of different portions of a given stream (figure 2) [42]. In both species, for example, the rostral portion of the upper jaw (e.g. premaxilla) and the frontonasal region are derived from the anterior portion of the mandibular stream, whereas the remainder of the upper jaw, the quadrate and nearly all of the lower jaw are derived from the posterior portion. Similarly, the hyoid stream contributes to the retroarticular process of the lower jaw (figure 2; ar) and the middle ear (stapes; not shown). The branchial stream makes no contribution to the skull proper in either species. The significance of the apparent difference in derivation of the maxillary bone between these species—from the anterior portion of the mandibular stream in axolotl but from the posterior portion of the same stream in chicken—is unclear. Anterior and posterior portions of each mandibular stream, as currently designated by the corresponding grafting protocols, may not represent equivalent cell populations in the two species, particularly in regards to migration pathways. Until these and other relevant details are resolved by additional mapping studies, especially in the axolotl, it is not possible to determine the developmental significance of this difference in derivation of the maxilla.

The only substantial difference between the fate maps for axolotl and chicken concerns the derivation of the frontal bone. In axolotl, as in mouse, CNC contributes to the frontal bone along its entire length, whereas in the chicken only the anterior portion of the frontal is derived from CNC. The parietal bone is derived exclusively from mesoderm in both the axolotl and chicken, as it is in the mouse.

### 3.3. Evolution of the tetrapod cranial vault

**Figure 3.**
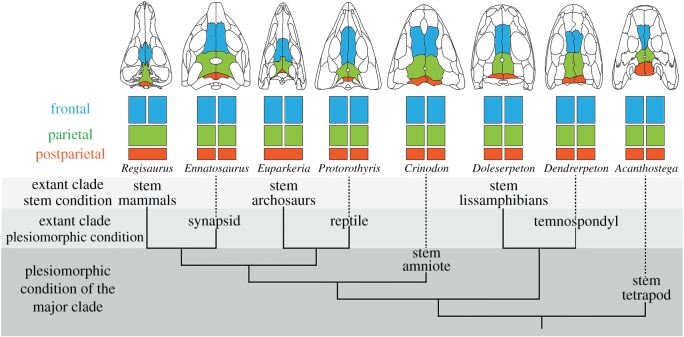
Bones of the cranial vault across extinct members of Tetrapoda mapped onto a composite phylogeny based on [43,44].

We conducted a literature review to document variation in cranial vault morphology among extinct tetrapods (see the electronic supplementary material, table S1). A nearly ubiquitous pattern, comprised of discrete frontals, parietals and postparietals, characterizes most taxa [1]. Optimizing all observed patterns on a consensus phylogeny of tetrapods reveals this compliment of bones to be present in stem members of each major lineage (figure 3). It thus represents a common, plesiomorphic pattern, both for these lineages and for tetrapods in general.

Alternate configurations of the cranial vault seen in both extinct and extant tetrapods constitute variations on this otherwise widespread pattern. Such variants include medial fusion of formerly paired parietals (e.g. some stem mammals) and medial fusion of formerly paired postparietals (e.g. some stem amniotes) [12,45]. Two independent instances of frontal–parietal fusion are seen in pachycephalosaur dinosaurs and stem anurans. Postparietal loss appears to have occurred multiple times, although several studies report the postparietal present in one or more species of several extant clades once thought to lack it, including salamanders, frogs, crocodylians and birds [10,11,14,46–49] (see also the electronic supplementary material, table S1).

## 4. Discussion

It is generally assumed that the embryonic origin of the skull is highly conserved among tetrapods [17]. Homologous bones, which typically are assigned the same names in different taxonomic groups, are thought to share the same embryonic origin with respect to their derivation from either CNC or mesoderm. These notions are challenged, however, by reported differences in the embryonic origin of the bone currently named the frontal between the chicken and mouse. As noted by several authors, these differences suggest either that embryonic origin is evolutionarily plastic or that homologies of bones of the cranial vault between birds and mammals, as implied by the names currently assigned to them, may be incorrect [12,16,20,33,38]. Indeed, one team of authors has attempted to resolve these differences by suggesting alternate names for the avian frontal and parietal [15].

Our data from axolotl provide evidence that CNC-derived versus mesoderm-derived portions of the skull are in general highly consistent among representatives of three tetrapod lineages—urodeles, birds and mammals. Based on this result, we suggest that these clades may retain an ancestral pattern of embryonic development of the osteocranium that was present in their common tetrapod ancestor, if not even earlier, in bony fishes [25]. Comparison of the detailed fate maps for axolotl and chicken reveals the high degree of similarity between these two distantly related species (figure 2). Even though axolotls and chickens have significantly different cranial morphologies and are separated by more than 300 million years of evolution, they share an almost identical pattern of embryonic derivation of the skull. This extreme similarity extends to the fates of individual CNC streams, and to a considerable extent even to the fates of regions within the first (mandibular) stream. Less detailed fate-map data for the mouse also are highly similar.

**Figure 4.**
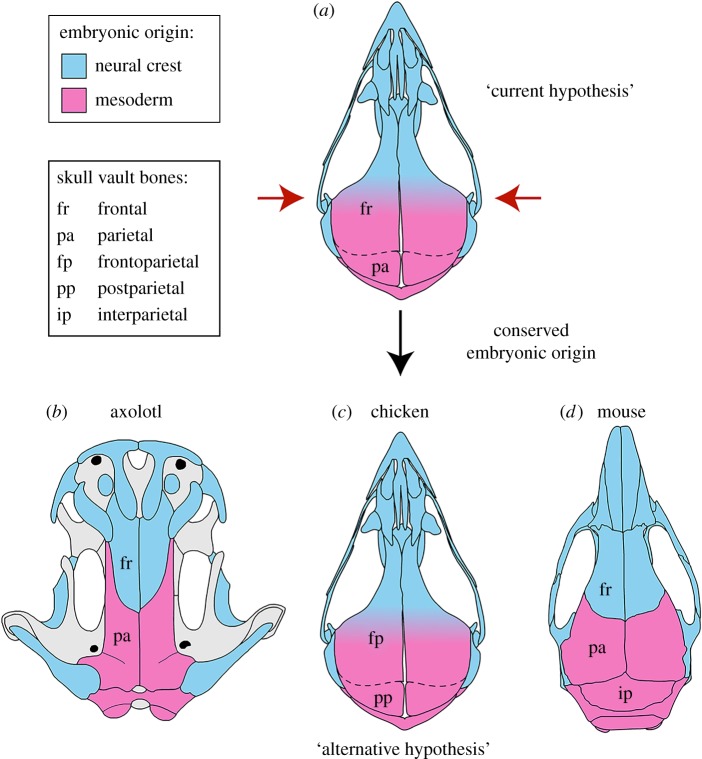
Comparison of CNC (blue) and mesoderm (pink) contributions to the cranial vault in the axolotl (this study), chicken [36] and mouse [20]. (*a*) According to the current hypothesis of homology of the cranial vault in birds, the CNC–mesoderm boundary lies within the frontal (fr; red arrows). This differs from both axolotl (*b*) and mouse (*d*), in which the boundary lies between the frontal and parietal (pa). Vault anatomy is further modified in adults of some avian species, e.g. chicken, in which the frontal and parietal fuse (dashed line). (*c*) In the alternative hypothesis, the avian ‘frontal’ is instead regarded as a frontoparietal (fp) and the ‘parietal’ as a postparietal (pp). In the adult mouse (and other mammals), the postparietal constitutes the median portion of the interparietal (ip).

These new data bolster the oft-stated contention that bones of the cranial vault in the chicken (indeed, in all birds) may be identified and named incorrectly with respect to cranial vault bones in other tetrapods [15,16,20,33]. Moreover, the apparent difference in the embryonic origin of bones of the cranial vault of birds relative to other tetrapods can be eliminated by interpreting the avian ‘frontal’ instead as a fused frontal and parietal, i.e. a frontoparietal, with an anterior (frontal) portion derived from CNC and a posterior (parietal) portion derived from mesoderm [15,16,20]. An attractive consequence of this alternative hypothesis of cranial vault homology is that the embryonic origin of bones with the same name, as well as the topologic position of both the CNC–mesoderm interface and the frontal–parietal boundary relative to the rest of the adult skull, are consistent among the chicken, mouse and axolotl (figure 4).

**Figure 5.**
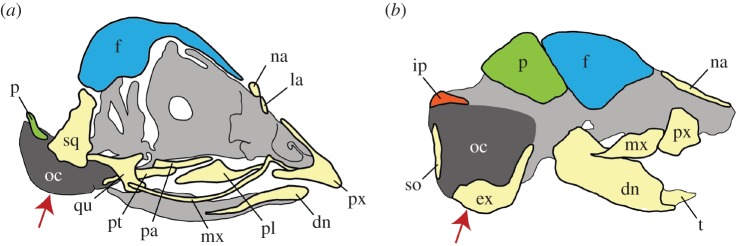
Illustrations of the developing skulls of the chicken (65 mm total length) and mouse (stage E15.5) embryos showing the topologic position of bones of the cranial vault relative to the chondrocranium (dark and light grey). Lateral views; anterior is to the right. (*a*) In the chicken, the currently named frontal (f, blue) forms considerably anterior to the otic capsule (oc, dark grey), whereas the parietal (p, green) forms at the level of the otic capsule, adjacent to the dorsal margin of the foramen magnum (red arrow). (*b*) In the mouse, both the frontal and the parietal form anterior to the otic capsule, at a comparable position to the avian frontal, whereas the interparietal (ip, red) forms at a comparable position to the avian parietal. dn, dentary; ex, exoccipital; la, lacrimal; mx, maxilla; na, nasal; pa, parasphenoid; pl, palatine; pt, pterygoid; px, premaxilla; qu, quadrate; so, supraoccipital; t, tooth. Diagrams in (*a*) and (*b*) are based on [50] and [51], respectively.

The topologic position of bones of the cranial vault with respect to conserved features of the chondrocranium early in development offers additional support for the alternative hypothesis. In mammals, which possess the full complement of vault bones, the frontal and parietal form considerably anterior to the otic capsule, whereas the much smaller interparietal forms next to the otic capsule along the dorsal margin of the foramen magnum ([50]; figure 5*b*). In birds, however, anterior and posterior portions of the currently named frontal form in comparable positions to the frontal and parietal of mammals, respectively, viz. well anterior to the otic capsule ([50]; figure 5*a*). Moreover, the currently named parietal forms initially as a tiny element at the posterior margin of the vault, next to the otic capsule and adjacent to the dorsal margin of the foramen magnum, thus resembling the interparietal of mammals. The same correspondence between vault bones and the chondrocranium is seen in living amphibians, including salamanders, frogs and caecilians, in which the frontal and parietal initially form well anterior to the otic capsule [50,52,53]. It also is the generalized condition for reptiles [1], including both *Sphenodon* and *Lacerta* [50], in which the frontal and parietal form adjacent to the taenia marginalis posterior, anterior to the otic capsule. By adopting the alternative nomenclature for the avian cranial vault, the topologic correspondence of individual vault bones to the chondrocranium is consistent across tetrapods.

Reinterpretation of the avian frontal as the product of fusion of previously discrete dermal bones is also supported by its pattern of ossification, particularly the number and location of ossification centres. In the chicken, each frontal bone forms from two discrete ossification centres arrayed rostrocaudally, which later fuse [49,54,55]. Although separate ossification centres within the frontal are not characteristic of all birds that have been examined developmentally [56,57], failure to observe a discrete ossification centre need not mean that the corresponding bone is absent [5,58]. Instead, it may be the result of observational limitations, e.g. key stages have not been examined [59], or any of several developmental phenomena, such as early fusion and/or rapid growth with the failure to differentiate discrete centres [5,50,58]. For example, frogs possess a frontoparietal, which evolved early in the anuran lineage [60]. In certain taxa, the bone forms from two discrete ossification centres that later fuse, but many other taxa lack discrete paired centres at any time during ontogeny [3,5,61]. Dugès distinguishes between two situations in which previously separate bones in an ancestor can become fused in a descendant, either without (‘fusion primordiale’) or with (‘fusion secondaire’) the presence of distinct ossification centres [50,62].

Continued use of the currently understood names, and thus implied homologies, of bones of the avian cranial vault implies acceptance of the fact that the frontal is of mixed embryonic origin (CNC and mesoderm) in the chicken, instead of an exclusive derivation from CNC as in the axolotl and mouse, and that the CNC–mesoderm interface is displaced anteriorly in birds relative to its location in urodeles and mammals (figure 4) [15]. Owing to technical limitations, cranial fate maps and genetic-labelling studies are available for very few model species—too few to rigorously assess the evolutionary variability of patterns of embryonic derivation of features we consider here. We, therefore, caution against drawing definitive conclusions based on the limited taxon sampling that exists at this time. Nevertheless, the pattern of embryonic origin of skull bones we describe in the axolotl appears to be highly conserved phylogenetically, even when comparing distantly related species such as zebrafish and amniotes [25,33,63]. The only exception documented to date is the clawed frog, *Xenopus laevis*, which has a very different pattern of CNC-derivation of skull bones in comparison with the one shared by mouse, chicken, axolotl and zebrafish [25]. The unique pattern in *Xenopus* probably evolved relatively recently, after the divergence of anurans and urodeles from their common ancestor, and may be a consequence of the extreme changes in cranial morphology and histology that characterize anuran metamorphosis. Alternatively, the data for *Xenopus* may itself challenge the currently understood homologies of cranial vault bones in anurans [3], which are not accepted by all authors [5–9].

The proposed reinterpretation of the homology of the frontal bone in birds discussed above has additional consequences, specifically regarding the homology of the bone posterior to it—the currently named parietal (figures 2 and 4*a*). This element occupies a position at the caudal end of the cranial vault, immediately anterior to the supraoccipital—the same position occupied by the postparietal in primitive tetrapods [1]. When present, the postparietal typically is paired, although the two bones are fused to form a single, median element in some taxa (figure 3). Considered the homologue of the element of the same name in tetrapodamorph fishes [64,65], the postparietal is a consistent component of the cranial vault throughout the early evolutionary history of all groups of tetrapods (figure 3). Indeed, the presence of a postparietal characterizes the plesiomorphic condition of each lineage of living tetrapods, including reptiles, and of tetrapods in general (figure 3) [1,12]. A corollary of the hypothesis that the avian ‘frontal’ is actually a frontoparietal is that the avian ‘parietal’ is more appropriately recognized as a postparietal. Is this reinterpretation justified?

There is an evolutionary trend towards loss of the postparietal in all lineages of living tetrapods [1,2]. The only widely recognized exception is synapsids (mammalian lineage), in which the postparietal constitutes the median portion of the interparietal (fused postparietals plus tabulars) [12]. The postparietal is generally considered lost in the archosaurian lineage, and reinterpretation of the avian ‘parietal’ as a postparietal seemingly conflicts with such claims [1,2]. However, a postparietal is present as a small median element in some stem archosaurs, including the archosauriforms *Proterosuchus*, *Erythrosuchus* and *Euparkeria* [66–68], the pseudosuchian *Gracilisuchus* [69,70] and possibly in members of Phytosauria [71,72] (but see [68]).

Additionally, nearly 60 years ago Romer noted that a small postparietal, which may fuse with the supraoccipital, is sometimes present in extant crocodylians, the sister taxon to birds [1]. More recently, Klembara identified a postparietal in an embryo of the extant crocodylian *Alligator* [14]. These claims suggest that the plesiomorphic condition for reptiles is retained in *Alligator,* and possibly other extant crocodylians, but also that the presence of a postparietal is obscured by its fusion to one or more adjacent bones early in development. Interestingly, there are several early accounts, now largely ignored, that allude to the presence of postparietal bones (typically termed interparietals) in various species of extant birds (e.g. [46,73]), although subsequent studies have failed to confirm such observations [12,59].

**Figure 6.**
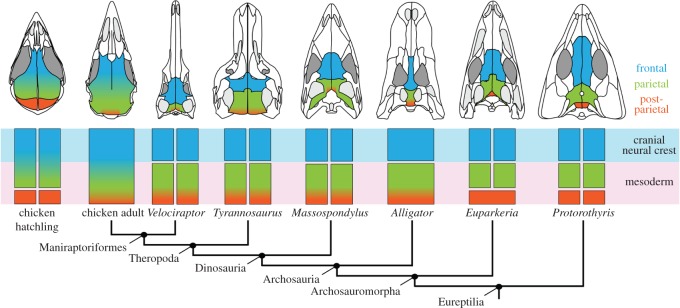
Alternative hypothesis for the evolution of the cranial vault in birds and other reptiles. All skulls are depicted in dorsal view; anterior is at the top. The plesiomorphic adult condition (*Protorothyris*) comprises paired frontals, parietals and postparietals. A prediction of the alternate hypothesis is that all three bones are retained in the more derived taxa, including birds, where they may fuse in various combinations. Composite phylogeny based on [76] and references therein.

Fusion of the postparietal to either the parietal anteriorly or the supraoccipital posteriorly is a common occurrence in mammals [12]. Such instances of postparietal ossification and fusion occur relatively late in cranial development [74], making it relatively easy to confirm the presence of the bone as a discrete element. If postparietal fusion occurred in extinct reptiles, then it probably took place much earlier in ontogeny [14], which would make the bone much more difficult to document in fossils. Consistent with this prediction, the bone that is here reinterpreted as the postparietal in the chicken fuses to the posterior, parietal portion of the frontoparietal at around 98 days posthatching [75]. If the alternative hypothesis is correct, then a postparietal may be present but fused to the parietal (or supraoccipital) in many, if not all, non-avian archosaurs, including dinosaurs (figure 6).

The medial portion of the synapsid interparietal is homologous with paired postparietals, whereas the mesoderm-derived tabulars constitute the lateral interparietal elements [12]. The proposed homology between the avian parietal and the postparietal of other tetrapods (including synapsids) is supported by experimental data that document a similar embryonic origin of these bones in birds and mammals: the bones are derived exclusively (chicken) or primarily (mouse) from mesoderm. Although initial studies in the mouse reported a CNC origin of the medial (postparietal) portion of the interparietal [18,77], subsequent studies have significantly downplayed any CNC contribution to this bone. Noden & Trainor [15], for example, restrict the contribution of CNC posterior to the parietal to the intercalvarial sutures. Morriss-Kay & Wilkie [78] describe the CNC contribution as ‘a patch of previously undetected CNC cells that emerge from the rostral hindbrain at E9.5 and insert into the dermis and form the central part of the interparietal bone’ ([78], p. 642). Finally, Richtsmeier & Flaherty [79] regard the interparietal as being derived mostly from mesoderm, except for a few cells of CNC origin. The possibility of a similarly scant contribution of CNC to the postparietal bone in the chicken remains to be evaluated [12], as comparable experiments involving the application of CNC molecular markers to assess embryonic origin have yet to be performed in any avian model.

If the ‘parietal’ of extant birds is instead the homologue of the postparietal of other tetrapods, then the bone may have increased considerably in size in comparison with the relatively small element seen in adult stem reptiles and archosauromorphs, and especially in early ontogenetic stages of extant crocodylians. A potential explanation for such size increase is the dramatic enlargement in the size of the brain that occurred in the maniraptoran lineage [80–82], which culminated in a level of encephalization (viz. brain volume relative to body size) in extant birds that is exceeded only by mammals [80,83,84]. In vertebrates generally, skull form and especially size are mediated to a considerable extent by both physical interactions with and molecular signals emerging from the developing brain [85–88]. We suggest that enlargement of the postparietal, along with many other defining features of the avian skull, is at least in part a response to evolution of the disproportionately large brain that they surround and support, in much the same way that increased encephalization has promoted evolutionary diversification of cranial morphology in mammals [89].

The search for evolutionary homologies is a defining feature of comparative biology. Hypotheses for the homology of skull bones among major groups of vertebrates have been in place for well over a hundred years (e.g. [90]), and the names given to most individual bones have been stable for much of that time [17]. Nevertheless, considerable uncertainty remains regarding the identity of particular elements in individual clades, ranging from ray-finned fishes to mammals [18,91]. Organismal development is an obvious and longstanding source of data for homology assessments, and while the use of ontogeny as the sole or even primary criterion for evaluating homology may not be justified in every instance [92,93]; features of development often play a critical role in differentiating between alternate hypotheses. Indeed, ontogenetic data have been central to several recent analyses that have clarified, and in some cases overturned, longstanding hypotheses of homology of cranial bones in anurans and mammals [12,18,94]. In other studies, alternative hypotheses of homology were proposed based on adult morphology [95,96]. Here, we have evaluated an alternative hypothesis for the homology of the cranial vault in birds proposed or implied by the results of several studies of the embryonic derivation of the cranial vault in amniote models. New ontogenetic data for the axolotl, a urodele, in combination with topologic comparisons and a phylogenetic analysis of character-state distribution among extant and extinct tetrapods, lends additional support to the alternative hypothesis.

## Supplementary Material

Supplementary Figure S1-Contribution of cranial neural crest streams and mesoderm to the bony skull of axolotl.

## Supplementary Material

Electronic Supplementary Materials: Transplantation method details. Supplementary Table S1-Summary of the composition of the cranial vault across tetrapods.

## Footnotes

1 Termed ‘interparietal’ in [15].

## Data accessibility

The cranial fate map for the axolotl and additional datasets supporting this article have been uploaded as part of the electronic supplementary material.
