# Supplementary figures and images for: Homology of the cranial vault in birds: new insights based on embryonic fate-mapping and character analysis
Source: R Soc Open Sci. 2016 Aug 10;3(8):160356. doi: 10.1098/rsos.160356 (PMC5108967; doi:10.1098/rsos.160356)

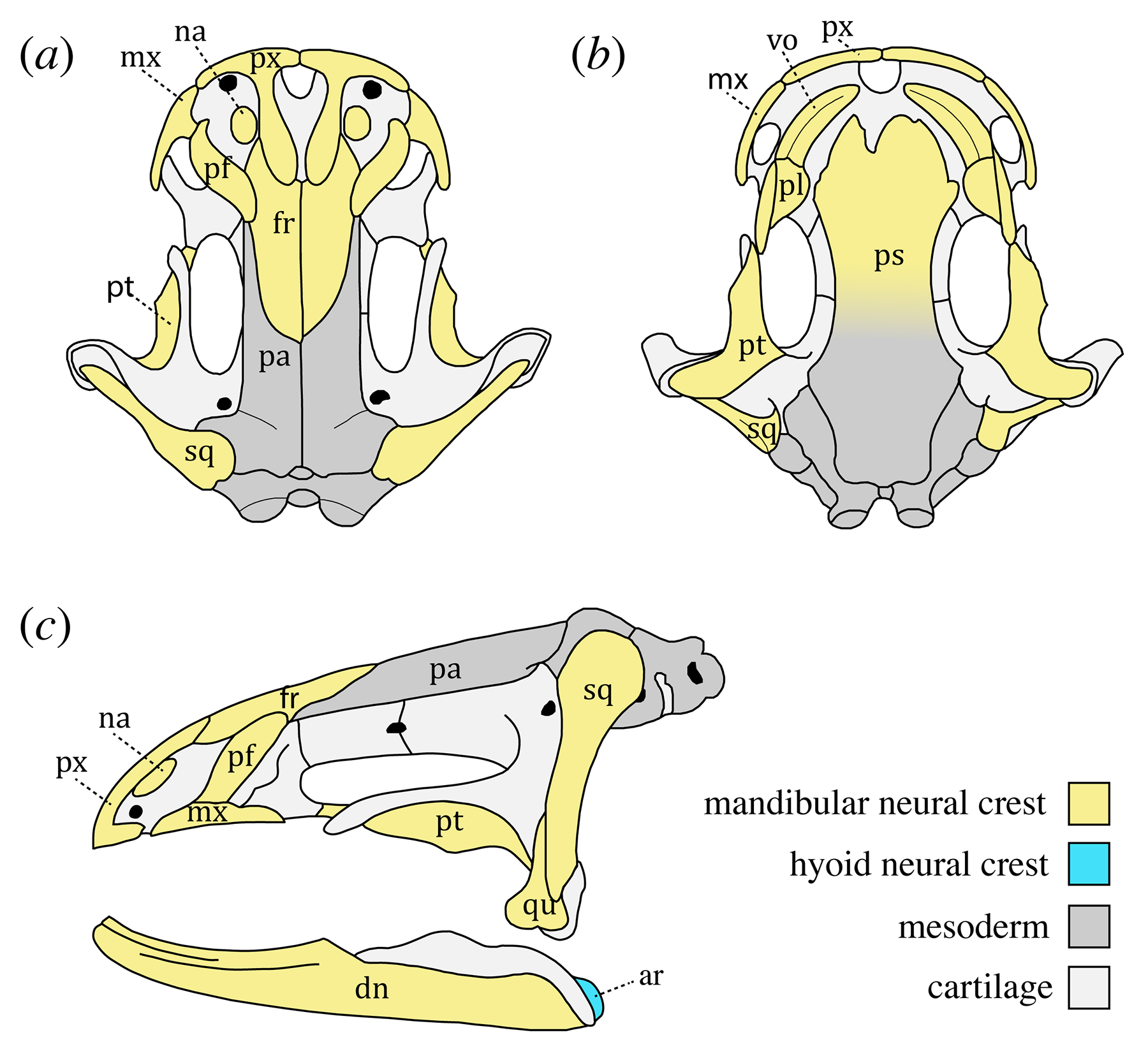

Supplement: Supplementary Figure S1-Contribution of cranial neural crest streams and mesoderm to the bony skull of axolotl. [file rsos160356supp1.tif]
