# Supplementary material for: Homology of the cranial vault in birds: new insights based on embryonic fate-mapping and character analysis
Source: R Soc Open Sci. 2016 Aug 10;3(8):160356. doi: 10.1098/rsos.160356 (PMC5108967; doi:10.1098/rsos.160356)
Supplement: Electronic Supplementary Materials: Transplantation method details. Supplementary Table S1-Summary of the composition of the cranial vault across tetrapods. [file rsos160356supp2.docx]

**Supplementary information:**

**Transplantation method details**

Neural fold transplantations were performed at neurula stages 15–19. Stages 16 and 17, when paired neural folds have not yet fused in the midline, were preferred. The neural fold was artificially divided into seven regions [1, 2]. A given region of donor neural fold was cut out by using tungsten needles and moved to the corresponding region of the stage-matched host, where it was inserted in place of an equal-sized piece of neural fold. Fluorescence microscopy was used to evaluate transplantation success and to identify which CNC stream was populated by transplanted cells as they migrated ventrally from the neural tube.

Cranial neural crest stream transplantations were performed between stages 20 and 25. The epidermis was cut and partly folded back to reveal underlying CNC streams. CNC cells at this stage are darkly pigmented and easy to distinguish from adjacent mesoderm. A segment of the mandibular, hyoid or branchial stream (see below for alternative neural crest stream terminology) was removed from the GFP-negative host embryo and replaced by a corresponding segment of the same stream of the GFP-positive donor. In younger embryos, at the onset of CNC migration, the transplant was taken from the neural fold and thus contained one CNC stream and a portion of the underlying neural tube. After transplantation, the host’s epidermis was folded back over the transplant. Migration of the GFP-positive cells was followed and documented by using fluorescence microscopy.

Cranial mesoderm transplantations were performed at stages 16–22. The cranial paraxial mesoderm was artificially divided into three regions. A small piece of donor mesoderm was cut out by using tungsten needles and moved to the corresponding location in the host, where it was inserted in place of an equal-sized piece of host mesoderm. Transplantations were evaluated by using fluorescence microscopy. To avoid possible contamination with CNC, transplantations were performed before ventrally migrating CNC cells reached the level of paraxial mesoderm.

**Histology and immunohistochemistry methods**

Infiltration with Optimal Cutting Temperature compound (OCT; Tissue Tek, Sakura Finetek, Tokyo, Japan) was performed by successive immersions in 15% sucrose, 30% sucrose, equal parts 30% sucrose and OCT, and pure OCT. Specimens were embedded in plastic molds in OCT, quick-frozen, and stored at -80°C. Serial transverse cryosections of 12–20 µm thickness were made with a Leica CM 3050S cryostat. Sections were collected onto VWR® Superfrost® Plus micro slides and stored at -20°C until further processing. Antibodies were applied to every other slide of the sectioning series to enhance the GFP signal. Sections on remaining slides (without antibody staining) served as a control. Sections were first blocked with 5% normal goat serum in PBS with Tween (PBST; Sigma-Aldrich, St. Louis, MO) for 2 h at room temperature. The primary antibody against GFP (rabbit anti-GFP, ab290; Abcam Antibodies, Cambridge, MA; 1:3000 in PBST + 5% normal goat serum) was then applied to the horizontal slides in a humidity chamber at 4°C. The next day, the slides were rinsed in PBS and in one final 5-min rinse in PBST before applying the secondary antibody (goat anti-rabbit Alexa 488; Molecular Probes, Eugene, OR; 1:1000 in PBST) onto the horizontal slides in a humidity chamber at 4°C. The next day, the slides were rinsed thoroughly in PBS. Alizarin red (0.5% in PBS) was applied to the horizontal slides for 3 min to stain calcified bone red. Subsequently, slides were rinsed in PBS and stained with DAPI (5 µg/ml; Sigma-Aldrich, St. Louis, MO). Finally, the slides were rinsed several times in PBS and mounted with a coverslip by using FluoromountG (Southern Biotech, Birmingham, AL). The sections were analyzed with a fluorescent microscope (Leica model DMRE equipped with B-filter; Leica, Banncokburn, IL). Images were taken and processed by using Openlab (Improvision, Boston, MA) or Volocity (PerkinElmer, Waltham, MA).

**Cranial neural crest stream terminology**

Cranial neural crest in all vertebrates, including agnathans, comprises three principal populations of migratory cells that emerge from different rostrocaudal positions along the developing brain [3-6]. Despite this basic similarity among taxa, these populations are frequently assigned different names by different authors in an attempt to convey the particular migratory pathway(s) followed and/or cranial region occupied by each population—features that may vary somewhat among species. We use “mandibular stream” in reference to the rostralmost of the three principal populations in the axolotl, whose cells migrate anterior and posterior to the eye and populate the first (mandibular) oropharyngeal arch [2]. It is equivalent to the “trigeminal” neural crest [3, 4], which may comprise distinct preoptic, postoptic and mandibular-arch streams [7, 8]. We use “hyoid stream” in reference to the second principal population, which populates the second (hyoid) arch. We use “branchial stream” in reference to the third population (“circumpharyngeal crest” [9]), which contributes to more posterior arches (in axolotl, arches 3–6).

**Supplementary figure**

**
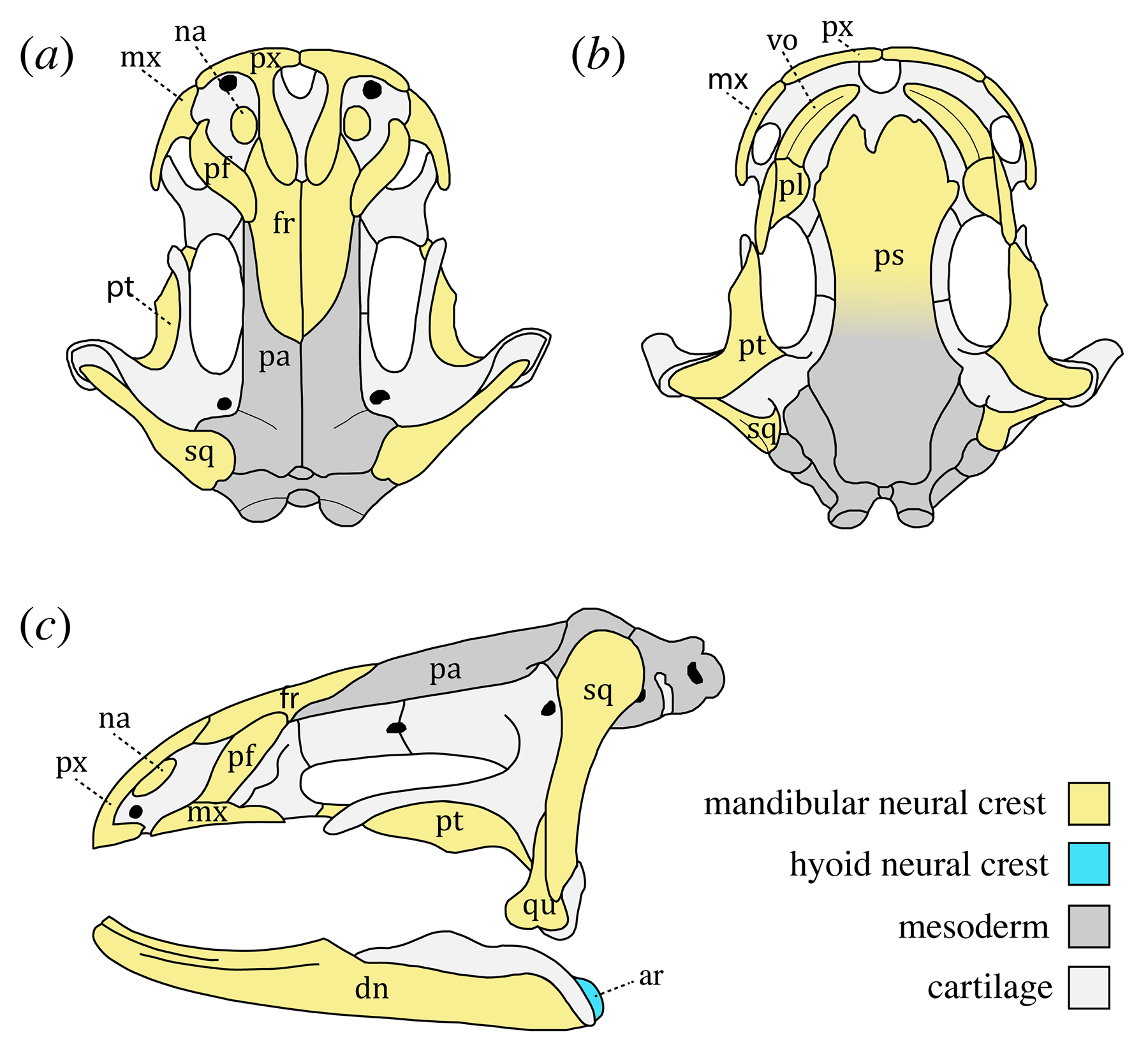
**

**Figure S1.** Contributions of individual cranial neural crest (CNC) streams and cranial mesoderm to the bony skull of the axolotl, seen in (*a*) dorsal, (*b*) ventral, and (*c*) lateral views. Most bones are derived from the mandibular stream (yellow). (*a,c*) Of the two principal skull-roofing bones, the frontal (fr) is derived from neural crest and the parietal (pa) from cranial mesoderm (dark grey). (*c*). The hyoid stream (blue) makes only minor contributions to the skull: the posterior (retroarticular) portion of the articular bone of the lower jaw (ar) and the stapes of the middle ear (not illustrated). The branchial stream does not contribute to the skull proper, but does contribute to the hyobranchial skeleton (not illustrated). Additional abbreviations: dn, dentary; mx, maxilla; na, nasal; pf, prefrontal; pl, palatine; ps, parasphenoid; pt, pterygoid; px, premaxilla; qu, quadrate; sq, squamosal; vo, vomer.

**Table S1. Summary of the composition of the cranial vault across Tetrapoda.**

Data for non-amniote taxa are taken from the analysis of Ruta and Coates [10], given its broad-spectrum sampling of taxa across widely agreed-upon clades. Amniote taxa are taken from several sources, since no single up-to-date phylogeny of amniotes currently exists. The reader is referred to Koyabu et al. [11] for a detailed account of cranial vault composition in the synapsid amniote lineage. For the reptilian lineage, a number of recent analyses were used to gather representative taxa for each major clade: parareptiles [12]; non-diapsid and early eureptiles [13]; non-neodiapsid diapsids [14]; non-archosauromorph neodiapsids [15]; and non-dinosaurian archosauromorphs [16], where phytosaurs were sampled from Stocker [17] and crocodylomorphs were sampled from Turner and Sertich [18]. Higher-level taxonomy is taken from associated sources; taxa in grey are individual branches that are not contained within the higher-level clade indicated for the taxa immediately preceding them in the list. Abbreviations: fr, frontal; pa, parietal; pp, postparietal.

| **Higher-level taxonomy** |  |  |  |  | **fr** | **pa** | **pp** | **Ref** |
| --- | --- | --- | --- | --- | --- | --- | --- | --- |
| Non-amniotes | Stem tetrapods |  |  | *Acanthostega* | ✓ | ✓ | ✓ | [19] |
|  |  |  |  | *Ichthyostega* | ✓ | ✓ | ✓ | [20] |
|  |  |  |  | *Tulerpeton* | ? | **?** | ? | [21] |
|  |  | Colosteidae |  | *Colosteus* | ✓ | ✓ | ✓ | [22] |
|  |  |  |  | *Greererpeton* | ✓ | ✓ | ✓ | [23] |
|  |  | Adelospondyli |  | *Adelogyrinus* | ✓ | ✓ | ✓ | [24] |
|  |  |  |  | *Adelospondylus* | ✓ | ✓ | ✓ | [25] |
|  |  |  |  | *Dolichopareias* | ✓ | ✓ | ✓ | [24] |
|  |  |  |  | *Crassigyrinus* | ✓ | ✓ | ✓ | [26] |
|  |  | Whatcheeriidae |  | *Ossinodus* | ✓ | ✓ | ✓ | [27] |
|  |  |  |  | *Pederpes* | ✓ | ✓ | ✓ | [28] |
|  |  |  |  | *Whatcheeria* | ✓ | ✓ | ✓ | [29] |
|  |  | Baphetidae |  | *Eucritta* | ✓ | ✓ | ✓ | [30] |
|  |  |  |  | *Baphetes* | ✓ | ✓ | ✓ | [31] |
|  |  |  |  | *Megalocephalus* | ✓ | ✓ | ✓ | [32] |
|  | Temnospondyli |  |  | *Edops* | ✓ | ✓ | ✓ | [33] |
|  |  |  |  | *Chenoprosopus* | ✓ | ✓ | ✓ | [34] |
|  |  |  |  | *Cochleosaurus* | ✓ | ✓ | ✓ | [35] |
|  |  |  |  | *Isodectes* | ✓ | ✓ | ✓ | [36] |
|  |  |  |  | *Trimerorhachis* | ✓ | ✓ | ✓ | [37] |
|  |  |  |  | *Balanerpeton* | ✓ | ✓ | ✓ | [38] |
|  |  |  |  | *Capetus* | ✓ | ✓ | ✓ | [39] |
|  |  |  |  | *Dendrerpeton* | ✓ | ✓ | ✓ | [40] |
|  |  |  |  | *Eryops* | ✓ | ✓ | ✓ | [41] |
|  |  | Dissorophoidea |  | *Acheloma* | ✓ | ✓ | ✓ | [42] |
|  |  |  |  | *Phonerpeton* | ✓ | ✓ | ✓ | [43] |
|  |  |  |  | *Ecolsonia* | ✓ | ✓ | ✓ | [44] |
|  |  |  |  | *Broiliellus* | ✓ | ✓ | ✓ | [45] |
|  |  |  |  | *Eoscopus* | ✓ | ✓ | ✓ | [46] |
|  |  |  |  | *Platyrhinops* | ✓ | ✓ | ✓ | [47] |
|  |  |  |  | *Micromelerpeton* | ✓ | ✓ | ✓ | [48] |
|  |  |  |  | *Apateon* | ✓ | ✓ | ✓ | [49] |
|  |  |  |  | *Amphibamus* | ✓ | ✓ | ✓ | [50] |
|  |  |  |  | *Doleserpeton* | ✓ | ✓ | ✓ | [51] |
|  |  |  | Lissamphibia | *Albanerpeton* | ✓ | ✓ |  | [52] |
|  |  |  |  | *Eocaecilia* | ✓ | ✓ |  | [53] |
|  |  |  |  | Caecilians | ✓ | ✓ |  | [54] |
|  |  |  |  | *Karaurus* | ✓ | ✓ |  | [55] |
|  |  |  |  | Salamanders | ✓ | ✓ |  | [54] |
|  |  |  |  | *Notobatrachus* | ✓ | ✓ |  | [56] |
|  |  |  |  | *Triadobatrachus* | ✓ | ✓ |  | [57] |
|  |  |  |  | Frogs | ✓ | ✓ |  | [54] |
|  |  |  |  | *Caerorhachis* | ✓ | ✓ | ✓ | [58] |
|  | Anthracosauria |  |  | *Silvanerpeton* | ✓ | ✓ | ✓ | [59] |
|  |  |  |  | *Eoherpeton* | ✓ | ✓ | ✓ | [60] |
|  |  |  |  | *Proterogyrinus* | ✓ | ✓ | ✓ | [61] |
|  |  |  |  | *Anthracosaurus* | ✓ | ✓ | ✓ | [62] |
|  |  |  |  | *Pholiderpeton* | ✓ | ✓ | ✓ | [63] |
|  | Gephyrostegidae |  |  | *Gephyrostegus* | ✓ | ✓ | ✓ | [64] |
|  | Seymouriamorpha |  |  | *Discosauriscus* | ✓ | ✓ | ✓ | [65] |
|  |  |  |  | *Seymouria* | ✓ | ✓ | ✓ | [66] |
|  |  |  |  | *Kotlassia* | ✓ | ✓ | ✓ | [67] |
|  |  |  |  | *Solenodonsaurus* | ✓ | ✓ | ✓ | [68] |
|  |  |  |  | *Westlothiana* | ✓ | ✓ | ✓ | [69] |
|  | Lepospondyli | Microsauria |  | *Tuditanus* | ✓ | ✓ | ✓ | [70] |
|  |  |  |  | *Asaphastera* | ✓ | ✓ | ✓ | [70] |
|  |  |  |  | *Saxonerpeton* | ✓ | ✓ | ✓ | [70] |
|  |  |  |  | *Batropetes* | ✓ | ✓ |  | [70] |
|  |  |  |  | *Carrolla* | ✓ | ✓ |  | [71] |
|  |  |  |  | *Quasicaecilia* | ✓ | ✓ |  | [70] |
|  |  |  |  | *Odonterpeton* | ✓ | ✓ | ✓ | [70] |
|  |  |  |  | *Hyoplesion* | ✓ | ✓ | ✓ | [70] |
|  |  |  |  | *Microbrachis* | ✓ | ✓ | ✓ | [70] |
|  |  |  |  | *Hapsidopareion* | ✓ | ✓ | ✓ | [70] |
|  |  |  |  | *Micraroter* | ✓ | ✓ | ✓ | [70] |
|  |  |  |  | *Pelodosotis* | ✓ | ✓ | ✓ | [70] |
|  |  |  |  | *Rhynchonkos* | ✓ | ✓ | ✓ | [70] |
|  |  |  |  | *Cardiocephalus* | ✓ | ✓ | ✓ | [70] |
|  |  |  |  | *Euryodus* | ✓ | ✓ | ✓ | [70] |
|  |  |  |  | *Pantylus* | ✓ | ✓ | ✓ | [70] |
|  |  | Lysorophia |  | *Brachydectes* | ✓ | ✓ | ✓ | [72] |
|  |  | Nectridea |  | *Scincosaurus* | ✓ | ✓ |  | [73] |
|  |  |  |  | *Keraterpeton* | ✓ | ✓ | ✓ | [73] |
|  |  |  |  | *Diplocaulus* | ✓ | ✓ | ✓ | [73] |
|  |  |  |  | *Diploceraspis* | ✓ | ✓ | ✓ | [73] |
|  |  |  |  | *Ptyonius* | ✓ | ✓ | ✓ | [73] |
|  |  |  |  | *Sauropleura* | ✓ | ✓ | ✓ | [73] |
|  |  |  |  | *Urocordylus* | ✓ | ✓ | ✓ | [73] |
|  |  | Aistopoda |  | *Lethiscus* | ✓ | ✓ | ✓ | [74] |
|  |  |  |  | *Oestocephalus* | ✓ | ✓ | ✓ | [74] |
|  |  | Phlegothontia |  | *Phlegothontia* | ✓ | ✓ |  | [74] |
|  | Diadectomorpha |  |  | *Tseajaia* | ✓ | ✓ | ✓ | [75] |
|  |  |  |  | *Limnoscelis* | ✓ | ✓ | ✓ | [76] |
|  |  |  |  | *Orobates* | ✓ | ✓ | ✓ | [77] |
|  |  |  |  | *Diadectes* | ✓ | ✓ | ✓ | [75] |
| Amniota | Non-diapsid reptiles | Parareptilia |  | *Macroleter* | ✓ | ✓ | ✓ | [78] |
|  |  |  |  | *Deltavjatia* | ✓ | ✓ | ✓ | [79] |
|  |  |  |  | *Procolophon* | ✓ | ✓ |  | [80] |
|  |  |  |  | *Nyctiphruretus* | ✓ | ✓ | ✓ | [81] |
|  |  |  |  | *Acleistorhinus* | ✓ | ✓ | ✓ | [82] |
|  |  |  |  | *Eunotosaurus* | ✓ | ✓ | ✓ | [83] |
|  |  |  |  | *Mesosaurus* | ✓ | ✓ | ✓ | [84] |
|  | Non-diapsid eureptiles |  |  | *Captorhinus* | ✓ | ✓ | ✓ | [85] |
|  |  |  |  | *Labidosaurikos* | ✓ | ✓ | ✓ | [86] |
|  |  |  |  | *Romeria* | ✓ | ✓ | ✓ | [87] |
|  |  |  |  | *Concordia* | ✓ | ✓ | ✓ | [88] |
|  |  |  |  | *Thuringothyris* | ✓ | ✓ | ✓ | [89] |
|  |  |  |  | *Paleothyris* | ✓ | ✓ | ✓ | [90] |
|  |  |  |  | *Hylonomus* | ✓ | ✓ | ✓ | [91] |
|  |  |  |  | *Protorothyris* | ✓ | ✓ | ✓ | [87] |
|  | Diapsida |  |  | *Araeoscelis* | ✓ | ✓ | ✓ | [92] |
|  |  |  |  | *Petrolacosaurus* | ✓ | ✓ | ✓ | [93] |
|  |  |  |  | *Kenyasaurus* | ? | ? | ? | [94] |
|  |  |  |  | *Lanthanolania* | ✓ | ? | ? | [95] |
|  |  |  |  | *Thadeosaurus* | ? | ? | ? | [96] |
|  |  |  |  | *Galesphyrus* | ? | ? | ? | [97] |
|  |  |  |  | *Youngina* | ✓ | ✓ | ✓ | [98] |
|  | Lepidosauromorpha |  |  | *Tangasaurus* | ? | ? | ? | [99] |
|  |  |  |  | *Acerosodontosaurus* | ✓ | ? | ? | [100] |
|  |  |  |  | *Hovasaurus* | ? | ✓ | ✓ | [101] |
|  |  |  |  | *Claudiosaurus* | ✓ | ✓ | ✓ | [96] |
|  |  |  |  | *Saurosternon* | ? | ? | ? | [102] |
|  |  |  |  | *Paliguana* | ✓ | ✓ | ✓ | [103] |
|  |  |  |  | *Palaeagama* | ✓ | ✓ | ? | [104] |
|  |  |  |  | *Coelurosauravus* | ✓ | ✓ |  | [105] |
|  |  |  |  | *Hupehsuchus* | ✓ | ✓ |  | [106] |
|  |  |  |  | *Ichthyosaurus* | ✓ | ✓ |  | [107] |
|  |  |  |  | *Askeptosaurus* | ✓ | ✓ |  | [108] |
|  |  |  |  | *Thalattosaurus* | ✓ | ✓ |  | [109] |
|  |  |  |  | *Clarazia* | ✓ | ✓ |  | [110] |
|  |  |  |  | *Kuehneosaurus* | ✓ | ✓ |  | [111] |
|  |  |  |  | *Placodus* | ✓ | ✓ |  | [112] |
|  |  |  |  | *Pachypleurosaurus* | ✓ | ✓ |  | [113] |
|  |  |  |  | *Yunguisaurus* | ✓ | ✓ |  | [114] |
|  |  |  |  | *Simosaurus* | ✓ | ✓ |  | [115] |
|  |  | Rhynchocephalia |  | *Clevosaurus* | ✓ | ✓ |  | [116] |
|  |  | Squamata |  | Lizards | ✓ | ✓ |  | [117] |
|  |  |  |  | Snakes | ✓ | ✓ |  | [117] |
|  | Archosauromorpha |  |  | *Champsosaurus* | ✓ | ✓ |  | [118] |
|  |  |  |  | *Tanystropheus* | ✓ | ✓ |  | [119] |
|  |  |  |  | *Prolacerta* | ✓ | ✓ |  | [98] |
|  |  |  |  | *Trilophosaurus* | ✓ | ✓ |  | [120] |
|  |  |  |  | *Rhynchosauria* | ✓ | ✓ |  | [121] |
|  |  | Archosauriformes |  | *Erythrosuchus* | ✓ | ✓ | ✓ | [122] |
|  |  |  |  | *Euparkeria* | ✓ | ✓ | ✓ | [123] |
|  |  |  |  | *Proterochampsa* | ✓ | ✓ |  | [124] |
|  |  |  | Phytosauria | *Paleorhinus* | ✓ | ✓ |  | [125] |
|  |  |  |  | *Smilosuchus* | ✓ | ✓ | ✓ | [126] |
|  |  |  |  | *Mystriosuchus* | ✓ | ✓ | ✓ | [127] |
|  |  |  | Ornithosuchidae | *Ornithosuchus* | ✓ | ✓ |  | [128] |
|  |  |  | Rauisuchia | *Rauisuchus* | ✓ | ✓ |  | [129] |
|  |  |  |  | *Saurosuchus* | ✓ | ✓ |  | [130] |
|  |  |  |  | *Arizonasaurus* | ✓ | ✓ |  | [131] |
|  |  |  | Aetosauridae | *Aetosaurus* | ✓ | ✓ |  | [132] |
|  |  |  |  | *Gracilisuchus* | ✓ | ✓ | ✓ | [133] |
|  |  |  | Crocodylomorpha | *Dibothrosuchus* | ✓ | ✓ |  | [134] |
|  |  |  |  | *Protosuchus* | ✓ | ✓ | ? | [135] |
|  |  |  |  | *Simosuchus* | ✓ | ✓ |  | [18] |
|  |  |  |  | *Notosuchus* | ✓ | ✓ | ✓ | [136] |
|  |  |  |  | *Dyrosaurus* | ✓ | ✓ |  | [137] |
|  |  |  |  | *Theriosuchus* | ✓ | ✓ |  | [138] |
|  |  |  |  | *Alligator* | ✓ | ✓ | ✓(?) | [139] |
|  |  |  |  | *Crocodylus* | ✓ | ✓ |  | [140] |
|  |  |  |  | *Gavialis* | ✓ | ✓ |  | [141] |
|  |  |  |  | *Lagerpeton* | ? | ? | ? | [142] |
|  |  |  |  | *Marasuchus* | ? | ? | ? | [143] |
|  |  |  |  | *Lewisuchus* | ✓ | ✓ |  | [144] |
|  |  |  |  | Pterosauria | ✓ | ✓ |  | [145] |
|  |  |  |  | Dinosauria | ✓ | ✓ |  | [145] |
|  |  |  |  | *Odontochelys* | ✓ | ✓ |  | [146] |
|  |  |  |  | *Proganochelys* | ✓ | ✓ |  | [147] |
|  |  |  |  | Turtles | ✓ | ✓ |  | [117] |
|  |  |  |  |  |  |  |  |  |

**Supplementary information references:**

[1] Cerny, R., Meulemans, D., Berger, J., Wilsch-Brauninger, M., Kurth, T., Bronner-Fraser, M. & Epperlein, H. 2004 Combined intrinsic and extrinsic influences pattern cranial neural crest migration and pharyngeal arch morphogenesis in axolotl. *Developmental Biology* **266**, 252-269.

[2] Epperlein, H., Meulemans, D., Bronner-Fraser, M., Steinbeisser, H. & Selleck, M. 2000 Analysis of cranial neural crest migratory pathways in axolotl using cell markers and transplantation. *Development* **127**, 2751-2761.

[3] Cerny, R., Lwigale, P., Ericsson, R., Meulemans, D., Epperlein, H. & Bronner-Fraser, M. 2004 Developmental origins and evolution of jaws: new interpretation of “maxillary” and “mandibular”. *Developmental Biology* **276**, 225-236.

[4] Kuratani, S. 2012 Evolution of the vertebrate jaw from developmental perspectives. *Evolution & Development* **14**, 76-92.

[5] Hall, B.K. 2009 *The neural crest and neural crest cells in vertebrate development and evolution*. 2nd ed. New York, NY, Springer.

[6] Le Douarin, N.M. & Kalcheim, C. 1999 *The neural crest*. 2nd ed. Cambridge, UK, Cambridge University Press.

[7] Kuratani, S., Nobusada, Y., Horigome, N. & Shigetani, Y. 2001 Embryology of the lamprey and evolution of the vertebrate jaw: insights from molecular and developmental perspectives. *Philosophical Transactions of the Royal Society of London. Series B, Biological Sciences* **356**, 15-32.

[8] Miyashita, T. 2015 Fishing for jaws in early vertebrate evolution: a new hypothesis of mandibular confinement. *Biological Reviews* **Early View**, 000-000.

[9] Hirasawa, T. & Kuratani, S. 2015 Evolution of the vertebrate skeleton: morphology, embryology, and development. *Zoological Letters* **1**, 1-17.

[10] Ruta, M. & Coates, M.I. 2007 Dates, nodes and character conflict: addressing the lissamphibian origins problem. *Journal of Systematic Palaeontology* **5**, 69-122.

[11] Koyabu, D., Maier, W. & Sanchez-Villagra, M.R. 2012 Paleontological and developmental evidence resolve the homology and dual embryonic origin of a mammalian skull bone, the interparietal. *Proceedings of the National Academy of Sciences of the United States of America* **109**, 14075-14080.

[12] Müller, J. & Tsuji, L.A. 2008 Impedance-matching hearing in Paleozoic reptiles: evidence of advanced sensory perception at an early stage of amniote evolution. *Public Library Online* **9**, 1-7.

[13] Muller, J. & Reisz, R.R. 2006 The phylogeny of early eureptiles: comparing parsimony and Bayesian approaches in the investigation of a basal fossil clade. *Systematic Biology* **55**, 503-511.

[14] Senter, P. 2004 Phylogeny of Drepanosauridae (Reptilia: Diapsida). *Journal of Systematic Palaeontology* **2**, 257-268.

[15] Bickelmann, C., Muller, J. & Reisz, R.R. 2009 The enigmatic diapsid *Acerosodontosaurus piveteaui* (Reptilia: Neodiapsida) from the Upper Permian of Madagascar and the paraphyly of "younginiform" reptiles. *Canadian Journal of Earth Sciences* **46**, 651-661.

[16] Brusatte, S.L., Benton, M.J., Desojo, J.B. & Langer, M.C. 2010 The higher-level phylogeny of Archosauria (Tetrapoda: Diapsida). *Journal of Systematic Palaeontology* **8**, 3-47.

[17] Stocker, M.R. 2012 A new phytosaur (Archosauriformes, Phytosauria) from the Lot's Wife beds (Sonsela Member) within the Chinle Formation (Upper Triassic) of Petrified Forest National Park, Arizona. *Journal of Vertebrate Paleontology* **32**, 573-586.

[18] Turner, A.H. & Sertich, J.J. 2010 Phylogenetic history of *Simosuchus clarki* (Crocodyliformes: Notosuchia) from the Late Cretaceous of Madagascar. *Journal of Vertebrate Paleontology* **30**, 177-236.

[19] Clack, J.A. 2002 The dermal skull roof of *Acanthostega gunnari*, an early tetrapod from the Late Devonian. *Transactions of the Royal Society of Edinburgh: Earth and Environmental Science* **93**, 17-33.

[20] Clack, J.A., Ahlberg, P.E., Finney, S.M., Alonso, P.D., Robinson, J. & Ketcham, R.A. 2003 A uniquely specialized ear in a very early tetrapod. *Nature* **425**, 65-69.

[21] Lebedev, O.A. & Coates, M.I. 1995 The postcranial skeleton of the Devonian tetrapod *Tulerpeton* curtum Lebedev. *Zoological Journal of the Linnean Society* **114**, 307-348.

[22] Hook, R.W. 1983 *Colosteus scutellatus* (Newberry), a primitive temnospondyl amphibian from the Middle Pennsylvanian of Linton, Ohio. *American Museum Novitates* **2770**, 1-41.

[23] Smithson, T.R. 1982 The cranial morphology of Greererpeton burkemorani Romer (Amphibia: Temnospondyli). *Zoological Journal of the Linnean Society* **76**, 29-90.

[24] Watson, D.M.S. 1929 The Carboniferous Amphibia of Scotland. *Palaeontologica Hungarica* **1**, 221-252.

[25] Carroll, R.L. 1967 An adelogyrinid lepospondyl amphibian from the Upper Carboniferous. *Canadian Journal of Zoology* **45**, 1-15.

[26] Panchen, A.L. 1985 On the amphibian *Crassigyrinus scoticus* Watson from the Carboniferous of Scotland. *Philos. Trans. R. Soc. Lond. Ser. B-Biol. Sci.* **309**, 505-568.

[27] Warren, A. 2007 New data on *Ossinodus pueri*, a stem tetrapod from the Early Carboniferous of Australia. *Journal of Vertebrate Paleontology* **27**, 850-862.

[28] Clack, J. & Finney, S. 2005 *Pederpes finneyae*, an articulated tetrapod from the Tournaisian of western Scotland. *Journal of Systematic Palaeontology* **2**, 311-346.

[29] Lombard, R.E. & Bolt, J.R. 1995 A new primitive tetrapod, *Whatcheeria deltae*, from the Lower Carboniferous of Iowa. *Palaeontology* **38**, 471-494.

[30] Clack, J. 2001 *Eucritta melanolimnetes* from the Early Carboniferous of Scotland, a stem tetrapod showing a mosaic of characteristics. *Earth and Environmental Science Transactions of the Royal Society of Edinburgh* **92**, 75-95.

[31] Milner, A.C., Milner, A.R. & Walsh, S.A. 2009 A new specimen of *Baphetes* from Nyrany, Czech Republic and the intrinsic relationships of the Baphetidae. *Acta Zoologica* **90**, 318-334.

[32] Beaumont, E.H. 1977 Cranial morphology of the Loxommatidae (Amphibia: Labyrinthodontia). *Philosophical Transactions of the Royal Society of London. Series B, Biological Sciences*, 29-101.

[33] Romer, A.S. & Witter, R.V. 1942 *Edops*, a primitive rhachitomous amphibian from the Texas red beds. *Journal of Geology* **50**, 925-960.

[34] Mehl, M. 1913 A description of *Chenoprosopus milleri*. *Publications of the Carnegie Institute, Washington* **186**, 11-16.

[35] Milner, A.R. & Sequeira, S.E. 2003 On a small *Cochleosaurus* described as a large Limnogyrinus (Amphibia, Temnospondyli) from the Upper Carboniferous of the Czech Republic. *Acta Palaeontologica Polonica* **48**, 143-147.

[36] Sequeira, S.E. 1998 The cranial morphology and taxonomy of the saurerpetontid *Isodectes obtusus* comb. nov.(Amphibia: Temnospondyli) from the Lower Permian of Texas. *Zoological Journal of the Linnean Society* **122**, 237-259.

[37] Williston, S. 1915 *Trimerorhachis*, a Permian temnospondyl amphibian. *The Journal of Geology* **1915**, 246-255.

[38] Milner, A. & Sequeira, S. 1993 The temnospondyl amphibians from the Visean of East Kirkton, West Lothian, Scotland. *Transactions of the Royal Society of Edinburgh: Earth Sciences* **84**, 331-361.

[39] Sequeira, S. & Milner, A. 1993 The temnospondyl amphibian *Capetus* from the Upper Carboniferous of the Czech Republic. *Palaeontology* **36**, 657-680.

[40] Holmes, R.B., Carroll, R.L. & Reisz, R.R. 1998 The first articulated skeleton of *Dendrerpeton acadianum* (Temnospondyli, Dendrerpetontidae) from the Lower Pennsylvanian locality of Joggins, Nova Scotia, and a review of its relationships. *Journal of Vertebrate Paleontology* **18**, 64-79.

[41] Sawin, H.J. 1941 The cranial anatomy of *Eryops megacephalus*. *Bulletin of the Museum of Comparative Zoology, Harvard* **88**, 407-463.

[42] Polley, B.P. & Reisz, R.R. 2011 A new Lower Permian trematopid (Temnospondyli: Dissorophoidea) from Richards Spur, Oklahoma. *Zoological Journal of the Linnean Society* **161**, 789-815.

[43] Dilkes, D.W. 1993 Biology and evolution of the nasal region in trematopid amphibians. *Palaeontology* **36**, 839-853.

[44] Berman, D.S., Reisz, R. & Eberth, D.A. 1985 *Ecolsonia cutlerensis*, an Early Permian dissorophid amphibian from the Cutler Formation of north-central New Mexico. *Circular of the New Mexico Bureau of Mines & Mineral Resources* **191**, 1-31.

[45] Williston, S.W. 1914 *Broiliellus*, a new genus of amphibians from the Permian of Texas. *The Journal of Geology* **1914**, 49-56.

[46] Daly, E. 1994 The Amphibamidae (Amphibia: Temnospondyli), with a description of a new genus from the Upper Pennsylvanian of Kansas. *University of Kansas Museum of Natural History, Miscellaneous Publications* **85**, 1-59.

[47] Clack, J.A. & Milner, A.R. 1994 *Platyrhinops* from the Upper Carboniferous of Linton and Nýřany and the family Amphibamidae (Amphibia: Temnospondyli). *Pollichia-Buch* **29**, 185-191.

[48] Boy, J.A. 1995 On the Micromelerpetontidae (Amphibia: Temnospondyli). 1. Morphology and palaeoecology of *Micromelerpeton credneri* (Lower Permian; soutwestern Germany. *Palaontologische Zeitschrift* **69**, 429-457.

[49] Frobisch, N.B. & Schoch, R.R. 2009 The largest specimen of *Apateon* and the life history pathway of neoteny in the Paleozoic temnospondyl family Branchiosauridae. *Fossil Record* **12**, 83-90.

[50] Gregory, J.T. 1950 Tetrapods of the Pennsylvanian nodules from Mazon Creek, Illinois. *American Journal of Science* **248**, 833-873.

[51] Bolt, J.R. 1969 Lissamphibian origins - possible protolissamphibian from Lower Permian of Oklahoma. *Science* **166**, 888-891.

[52] McGowan, G.J. 2002 Albanerpetontid amphibians from the Lower Cretaceous of Spain and Italy: a description and reconsideration of their systematics. *Zoological Journal of the Linnean Society* **135**, 1-32.

[53] Jenkins Jr., F.A. & Walsh, D.M. 1993 An Early Jurassic caecilian with limbs. *Nature* **365**, 246-250.

[54] Duellman, W.E. & Trueb, L. 1994 *Biology of Amphibians*. Baltimore and London, The John Hopkins University Press; 670 p.

[55] Ivakhnenko, M. 1978 Urodeles from the Triassic and Jurassic of Soviet Central Asia. *Palaeontologicheski Zhurnal* **1978**, 84-89 [in Russian].

[56] Baez, A.M. & Nicoli, L. 2008 A new species of *Notobatrachus* (Amphibia, Salientia) from the Middle Jurassic of northwestern Patagonia. *Journal Information* **82**, 372-376.

[57] Rage, J.-C. & Roček, Z. 1989 Redescription of *Triadobatrachus massinoti* (Piveteau, 1936) an anuran amphibian from the Early Triassic. *Palaeontographica* **206**, 1-16.

[58] Ruta, M., Milner, A.R. & Coates, M.I. 2001 The tetrapod *Caerorhachis bairdi* Holmes and Carroll from the Lower Carboniferous of Scotland. *Earth and Environmental Science Transactions of the Royal Society of Edinburgh* **92**, 229-261.

[59] Ruta, M. & Clack, J.A. 2006 A review of *Silvanerpeton miripedes*, a stem amniote from the Lower Carboniferous of East Kirkton, West Lothian, Scotland. *Transactions of the Royal Society of Edinburgh: Earth Sciences* **97**, 31-63.

[60] Smithson, T. 1985 The morphology and relationships of the Carboniferous amphibian *Eoherpeton watsoni* Panchen. *Zoological Journal of the Linnean Society* **85**, 317-410.

[61] Holmes, R. 1984 The Carboniferous amphibian *Proterogyrinus scheelei* Romer, and the early evolution of tetrapods. *Philosophical Transactions of the Royal Society of London. Series B, Biological Sciences* **306**, 431-524.

[62] Panchen, A.L. 1977 On *Anthracosaurus russelli* Huxley (Amphibia: Labyrinthodontia) and the family Anthracosauridae. *Philosophical Transactions of the Royal Society of London. Series B, Biological Sciences* **279**, 447-512.

[63] Clack, J.A. 1987 *Pholiderpeton scutigerum* Huxley, an amphibian from the Yorkshire Coal Measures. *Philosophical Transactions of the Royal Society of London. Series B, Biological Sciences* **318**, 1-107.

[64] Klembara, J., Clack, J.A., Milner, A.R. & Ruta, M. 2014 Cranial anatomy, ontogeny, and relationships of the Late Carboniferous tetrapod *Gephyrostegus bohemicus* Jaekel, 1902. *Journal of Vertebrate Paleontology* **34**, 774-792.

[65] Klembara, J. 1994 The sutural pattern of skull-roof bones in Lower Permian *Discosauriscus austriacus* from Moravia. *Lethaia* **27**, 85-95.

[66] Berman, D.S., Reisz, R.R. & Eberth, D.A. 1987 *Seymouria sanjuanensis* (Amphibia, Batrachosauria) from the Lower Permian Cutler Formation of north-central New Mexico and the occurrence of sexual dimorphism in that genus questioned. *Canadian Journal of Earth Sciences* **24**, 1769-1784.

[67] Bystrow, A.P. 1944 *Kotlassia prima* Amalitzky. *Geological Society of American Bulletin* **55**, 379-416.

[68] Laurin, M. & Reisz, R.R. 1999 A new study of *Solenodonsaurus janenschi*, and a reconsideration of amniote origins and stegocephalian evolution. *Canadian Journal of Earth Sciences* **36**, 1239-1255.

[69] Smithson, T.R. & Rolfe, W.D.I. 1990 *Westlothiana* gen. nov.: naming the easliest known reptile. *Scottish Journal of Geology* **26**, 137-138.

[70] Carroll, R.L. 1998 Order Microsauria. In *Handbuch der Paläoherpetologie* (ed. P. Wellnhofer), pp. 1-71. München, Verlag Dr. Friedrich Pfeil.

[71] Maddin, H.C., Olori, J.C. & Anderson, J.S. 2011 A redescription of *Carrolla craddocki* (Lepospondyli: Brachystelechidae) based on high-resolution CT, and the impacts of miniaturization and fossoriality on morphology. *Journal of Morphology* **272**, 722-743.

[72] Wellstead, C.F. 1998 Order Lysorophia. In *Handbuch der Paläoherpetologie* (ed. P. Wellnhofer), pp. 133-148. München, Verlag Dr. Friedrich Pfeil.

[73] Bossy, K.A. & Milner, A.C. 1998 Order Nectridea. In *Handbuch der Paläoherpetologie* (ed. P. Wellnhofer), pp. 73-132. München, Verlag Dr. Friedrich Pfeil.

[74] Carroll, R.L. 1998 Order Aïstopoda. In *Handbuch der Paläoherpetologie* (ed. P. Wellnhofer), pp. 163-182. München, Verlag Dr. Friedrich Pfeil.

[75] Berman, D.S., Sumida, S.S. & Lombard, R.E. 1992 Reinterpretation of the temporal and occipital regions in *Diadectes* and the relationships of diadectomorphs. *Journal of Paleontology* **66**, 481-499.

[76] Romer, A.S. 1946 The primitive reptile *Limnoscelis* restudied. *American Journal of Science* **244**, 149-188.

[77] Berman, D.S., Henrici, A.C., Kissel, R.A., Sumida, S.S. & Martens, T. 2004 A new diadectid (Diadectomorpha), *Orobates pabsti*, from the Early Permian of central Germany. *Bulletins of Carnegie Museum of Natural History* **35**, 1-36.

[78] Tsuji, L.A. 2006 Cranial anatomy and phylogenetic affinities of the Permian parareptile *Macroleter poezicus*. *Journal of Vertebrate Paleontology* **26**, 849-865.

[79] Tsuji, L.A. 2013 Anatomy, cranial ontogeny and phylogenetic relationships of the pareiasaur *Deltavjatia rossicus* from the Late Permian of central Russia. *Earth and Environmental Science Transactions of the Royal Society of Edinburgh* **104**, 81-122.

[80] Carroll, R.L. & Lindsay, W. 1985 Cranial anatomy of the primitive reptile *Procolophon*. *Canadian Journal of Earth Sciences* **22**, 1571-1587.

[81] Saila, L.K. 2010 The phylogenetic position of *Nyctiphruretus acudens*, a parareptile from the Permian of Russia. *Journal of Iberian Geology* **36**, 123-143.

[82] Daly, E. 1969 A new procolophonoid reptile from the Lower Permian of Oklahoma. *Journal of Paleontology* **43**, 676-687.

[83] Gow, C.E. 1997 A reassessment of *Eunotosaurus africanus* Seeley (Amniota: Parareptilia). **34**, 33-42.

[84] Pineiro, G., Ferigolo, J., Ramos, A. & Laurin, M. 2012 Cranial morphology of the Early Permian mesosaurid *Mesosaurus tenuidens* and the evolution of the lower temporal fenestration reassessed. *Comptes Rendus Palevol* **11**, 379-391.

[85] Fox, R.C. & Bowman, M.C. 1966 Osteology and relationships of *Captorhinus aguti* (Cope)(Reptilia: Captorhinomorpha). **Article 11**, 1-79.

[86] MacLean 3rd, W.P. 1970 The braincase of *Labidosaurikos* (a Permian captorhinomorph reptile). *Journal of Paleontology* **44**, 458-463.

[87] Clark, J. & Carroll, R.L. 1973 Romeriid reptiles from the Lower Perrnian. *Bulletin of the Museum of Comparative Zoology, Harvard* **144**, 535-406.

[88] Muller, J. & Reisz, R.R. 2005 An early captorhinid reptile (Amniota, Eureptilia) from the Upper Carboniferous of Hamilton, Kansas. *Journal of Vertebrate Paleontology* **25**, 561-568.

[89] Muller, J., Berman, D.S., Henrici, A.C., Martens, T. & Sumida, S.S. 2006 The basal reptile *Thuringothyris mahlendorffae* (Amniota: Eureptilia) from the Lower Permian of Germany. *Journal Information* **80**.

[90] Carroll, R.L. 1969 A Middle Pennsylvanian captorhinomorph, and the interrelationships of primitive reptiles. *Journal of Paleontology* **43**, 151-170.

[91] Carroll, R.L. 1963 The earliest reptiles. *Journal of the Linnean Society (Zool)* **45**, 61-83.

[92] Vaughn, P.P. 1955 The Permian reptile *Araeoscelis* restudied. *Bulletin of the Museum of Comparative Zoology, Harvard* **113**, 303-467.

[93] Reisz, R.R. 1981 A diapsid reptile from the Pennsylvanian of Kansas. *University of Kansas Museum of Natural History* **Publication 7**, 1-74.

[94] Harris, J.M. & Carroll, R.L. 1977 *Kenyasaurus*, a new eosuchian reptile from the Early Triassic of Kenya. *Journal of Paleontology* **51**, 139-149.

[95] Modesto, S.P. & Reisz, R.R. 2002 An enigmatic new diapsid reptile from the Upper Permian of Eastern Europe. *Journal of Vertebrate Paleontology* **22**, 851-855.

[96] Carroll, R.L. 1981 Plesiosaur ancestors from the Upper Permian of Madagascar. *Philosophical Transactions of the Royal Society of London. Series B, Biological Sciences* **293**, 315-383.

[97] Carroll, R.L. 1976 *Galesphyrus capensis*, a younginid eosuchian from South Africa. *Annals of the South African Museum* **72**, 59-68.

[98] Gow, C.E. 1975 The morphology and relationships of *Youngina capensis* Broom and *Prolacerta broomi* Parrington. *Palaeontologia Africana* **18**, 89-131.

[99] Haughton, S.H. 1924 On reptilian remains from the Karroo beds of East Africa. *Quarterly Journal of the Geological Society of London* **137**, 1-11.

[100] Currie, P.J. 1980 A new younginid (Reptilia: Eosuchia) from the Upper Permian of Madagascar. *Canadian Journal of Earth Sciences* **17**, 500-511.

[101] Currie, P.J. 1981 *Hovasaurus boulei*, an aquatic eosuchian from the Upper Permian of Madagascar. *Palaeontologia Africana* **24**, 99-168.

[102] Huxley, T. 1868 I.--On *Saurosternon Bainii*, and *Pristerodon McKay*i, Two New Fossil Lacertilian Reptiles from South Africa. *Geological Magazine* **5**, 201-205.

[103] Carroll, R.L. 1982 A short limbed lizard from the Lystrosaurus zone (Lower Triassic) of South Africa. *Journal of Paleontology* **56**, 183-190.

[104] Carroll, R.L. 1975 Permo-Triassic" lizards" from the Karroo. *Palaeontologia Africana* **18**, 71-87.

[105] Evans, S.E. & Haubold, H. 1987 A review of the Upper Permian genera *Coelurosauravus*, *Weigeltisaurus* and *Gracilisaurus* (Reptilia: Diapsida). *Zoological Journal of the Linnean Society* **90**, 275-303.

[106] Carroll, R.L. & Zhi-Ming, D. 1991 *Hupehsuchus*, an enigmatic aquatic reptile from the Triassic of China, and the problem of establishing relationships. *Philosophical Transactions of the Royal Society of London. Series B: Biological Sciences* **331**, 131-153.

[107] Sollas, W.J. 1918 The skull of *Ichthyosaurus*, studied in serial sections. *Philosophical Transactions of the Royal Society of London. Series B, Containing Papers of a Biological Character* **208**, 63-126.

[108] Muller, J. 2005 The anatomy of *Askeptosaurus italicus* from the Middle Triassic of Monte San Giorgio and the interrelationships of thalattosaurs (Reptilia, Diapsida). *Canadian Journal of Earth Sciences* **42**, 1347-1367.

[109] Merrian, J.C. 1904 A new marine reptile from the Triassic of California. *University of California Publications, Bulletin of the Department of Geology* **3**, 419-421.

[110] Rieppel, O. 1987 *Clarazia* and *Hescheleria*; a reinvestigation of two problematic reptiles from the Middle Triassic of Monte San Giorgio (Switzerland). *Palaeontographica* **195**, 101-129.

[111] Robinson, P.L. 1962 Gliding lizards from the Upper Keuper of Great Britain. *Proceedings of the Geological Society of London* **1601**, 137-146.

[112] Sues, H.-D. 1987 On the skull of *Placodus gigas* and the relationships of the Placodontia. *Journal of Vertebrate Paleontology* **7**, 138-144.

[113] Carroll, R.L. & Gaskill, P. 1985 The nothosaur *Pachypleurosaurus* and the origin of plesiosaurs. *Philosophical Transactions of the Royal Society of London. Series B, Biological Sciences* **309**, 343-393.

[114] Cheng, Y.-N., Sato, T., Wu, X.-C. & Li, C. 2006 First complete pistosauroid from the Triassic of China. *Journal of Vertebrate Paleontology* **26**, 501-504.

[115] Rieppel, O. 1994 Osteology of *Simosaurus gaillardoti* and the relationships of stem-group Sauropterygia. *Fieldiana: Geology* **28**, 1-85.

[116] Swinton, W.W. 1939 A new Triassic rhynchocephalian from Gloucestershire. *Annals of the Magazine of Natural History* **11**, 591-594.

[117] Zug, G.R., Vitt, L.J. & Caldwell, J.P. 2001 *Herpetology: An Introductory Biology of Amphibians and Reptiles*. San Diego, Academic Press.

[118] Brown, B. 1905 The osteology of *Champsosaurus* Cope. *Memoirs of the American Museum of Natural History* **9**, 3-26.

[119] Nosotti, S. 2007 *Tanystropheus longobardicus* (Reptilia, Protorosauria): re-interpretation of the anatomy based on new specimens from the Middle Triassic of Besano (Lombardy, northern Italy). *Memoirie della Societa Italiana di Scienze Naturali e del Museo Civico di Storia Naturale di Milano* **35**, 1-88.

[120] Heckert, A.B., Lucas, S.G., Rinehart, L.F., Spielmann, J.A., Hunt, A.P. & Kahle, R. 2006 Revision of the archosauromorph reptile *Trilophosaurus*, with a description of the first skull of *Trilophosaurus jacobsi*, from the Upper Triassic Chinle Group, West Texas, USA. *Palaeontology* **49**, 621-640.

[121] Benton, M.J. 1990 The species of *Rhynchosaurus*, a rhynchosaur (Reptilia, Diapsida) from the Middle Triassic of England. *Philosophical Transactions of the Royal Society of London. Series B, Biological Sciences* **328**, 213-306.

[122] Gower, D.J. 1997 The braincase of the early archosaurian reptile *Erythrosuchus africanus*. *Journal of Zoology* **242**, 557-576.

[123] Ewer, R.F. 1965 The anatomy of the thecodont reptile *Euparkeria capensis* Broom. *Philosophical Transactions of the Royal Society of London. Series B, Biological Sciences* **248**, 379-435.

[124] Dilkes, D. & Arcucci, A. 2012 *Proterochampsa barrionuevoi* (Archosauriformes: Proterochampsia) from the Late Triassic (Carnian) of Argentina and a phylogenetic analysis of Proterochampsia. *Palaeontology* **55**, 853-885.

[125] Lees, J. 1907 The skull of *Paleorhinus*: a Wyoming phytosaur. *The Journal of Geology* **15**, 121-151.

[126] Camp, C.L. 1930 A study of the phytosaurs with new material from western North America. *Memoirs of the University of California* **10**, 1-161.

[127] Hungerbühler, A. 2002 The Late Triassic phytosaur *Mystriosuchus westphali*, with a revision of the genus. *Palaeontology* **45**, 377-418.

[128] Walker, A.D. 1964 Triassic reptiles from the Elgin area: *Ornithosuchus* and the origin of carnosaurs. *Philosophical Transactions of the Royal Society of London. Series B, Biological Sciences* **248**, 53-134.

[129] Huene, F. 1942 *Die fossilen Reptilien des sudamerikanischen Gondwanalandes*. Munchen, C. H. Beck; 332 p.

[130] Alcober, O. 2000 Redescription of the skull of *Saurosuchus galilei* (Archosauria: Rauisuchidae). *Journal of Vertebrate Paleontology* **20**, 302-316.

[131] Nesbitt, S.J. 2005 Osteology of the Middle Triassic pseudosuchian archosaur *Arizonasaurus babbitti*. *Historical Biology* **17**, 19-47.

[132] Huene, F. 1920 Osteologie von *Aetosaurus ferratus* O. FRAAS. *Acta Zoologica* **1**, 465-491.

[133] Romer, A.S. 1972 The Chanares (Argentina) Triassic reptile fauna XIII: An early ornithosuchid pseudosuchian, *Gracilisuchus stipanicicorum*. Gen. et Sp. nov. *Breviora* **389**, 1-24.

[134] Wu, X.-C. & Chatterjee, S. 1993 *Dibothrosuchus elaphros*, a crocodylomorph from the Lower Jurassic of China and the phylogeny of the Sphenosuchia. *Journal of Vertebrate Paleontology* **13**, 58-89.

[135] Gow, C. 2000 The skull of *Protosuchus haughtoni*, an Early Jurassic crocodyliform from southern Africa. *Journal of Vertebrate Paleontology* **20**, 49-56.

[136] Andrade, M.d. & Bertini, R.J. 2008 Morphological and anatomical observations about *Mariliasuchus amarali* and *Notosuchus terrestris* (Mesoeucrocodylia) and their relationships with other South American notosuchians. *Arquivos do Museu Nacional* **66**, 5-62.

[137] Jouve, S.p. 2005 A new description of the skull of *Dyrosaurus phosphaticus* (Thomas, 1893)(Mesoeucrocodylia: Dyrosauridae) from the Lower Eocene of North Africa. *Canadian Journal of Earth Sciences* **42**, 323-337.

[138] Schwarz, D. & Salisbury, S.W. 2005 A new species of *Theriosuchus* (Atoposauridae, Crocodylomorpha) from the Late Jurassic (Kimmeridgian) of Guimarota, Portugal. *Geobios* **38**, 779-802.

[139] Klembara, J. 2001 Postparietal and prehatching ontogeny of the supraoccipital in *Alligator mississippiensis* (Archosauria, Crocodylia). *Journal of Morphology* **249**, 147-153.

[140] Iordansky, N.N. 1973 The skull of the Crocodilia. In *Biology of the Reptilia* (eds. C. Gans & T.E. Parsons), pp. 201-262. London, Academic Press.

[141] Martin, J.E., Buffetaut, E., Naksri, W., Lauprasert, K. & Claude, J. 2012 *Gavialis* from the Pleistocene of Thailand and Its Relevance for Drainage Connections from India to Java. *PLOS ONE* **7**, e44541.

[142] Sereno, P.C. & Arcucci, A.B. 1994 Dinosaurian precursors from the Middle Triassic of Argentina: *Lagerpeton chanarensis*. *Journal of Vertebrate Paleontology* **13**, 385-399.

[143] Sereno, P.C. & Arcucci, A.B. 1994 Dinosaurian precursors from the Middle Triassic of Argentina: *Marasuchus lilloensis*, gen. nov. *Journal of Vertebrate Paleontology* **14**, 53-73.

[144] Romer, A.S. 1972 The Chanares (Argentina) Triassic reptile fauna. XIV. *Lewisuchus admixtus* gen. et sp. nov., a further thecodont from the Chanares beds. *Breviora* **390**, 1-13.

[145] Romer, A.S. 1956 *Osteology of the reptiles*. Chicago and London, The University of Chicago Press; 772 p.

[146] Li, C., Wu, X.-C., Rieppel, O., Wang, L.-T. & Zhao, L.-J. 2008 An ancestral turtle from the Late Triassic of southwestern China. *Nature* **456**, 497-501.

[147] Gaffney, E.S. & Meeker, L.J. 1983 Skull morphology of the oldest turtles: A preliminary description of *Proganochelys quenstedti*. *Journal of Vertebrate Paleontology* **3**, 25-28.
